# Factors associated with mortality in rear-seated adult passengers involved in fatal motor vehicle crashes on US roadways

**DOI:** 10.1186/s40621-015-0036-5

**Published:** 2015-03-19

**Authors:** Eli Raneses, Joyce C Pressley

**Affiliations:** 1 Department of Epidemiology, Columbia University, 722 West 168th St., New York, NY 10032 USA; 2 Department of Health Policy and Management, Columbia University, 722 West 168th St., New York, NY 10032 USA; 3 The Center for Injury Epidemiology and Prevention at Columbia, Columbia University, 722 West 168th St., New York, NY 10032 USA; 4 Mailman School of Public Health, Columbia University, 722 West 168th St., New York, NY 10032 USA

**Keywords:** Motor vehicle crashes, Rear-seated passenger mortality, Side crash test ratings, Seat belts, Point of impact, Seat position, Mortality, Elderly passengers

## Abstract

**Background:**

Recent efforts to pass rear seat belt laws for adults have been hampered by large gaps in the scientific literature. This study examines driver, vehicle, crash, and passenger characteristics associated with mortality in rear-seated adult passengers.

**Methods:**

The Fatality Analysis Reporting System (FARS) 2010 to 2011 was used to examine motor vehicle occupant mortality in rear-seated adult passengers 18 years and older. Side crash vehicle safety ratings were assessed in a subset analysis of vehicles struck on the same side as the rear-seated passenger. Multilevel logistic regression models used SAS GLIMMIX.

**Results:**

Of the 7,229 rear-seated adult passengers, 2,091 (28.9%) died. Multivariable predictors of increased mortality were advancing passenger age, younger driver age, excessive speed, ejection, being unbelted, rear impact, and same-side crash. Belt use was associated with a 67.0% reduction in total mortality. Despite this, belt wearing was low (48.1%) and differed by seating position, with less than one third of middle-seated passengers belted. Multivariable analysis showed mortality to be nearly three times higher in same-side crashes than other impact locations (odds ratio (OR) = 2.76, 2.22, 3.44). In a multivariable subpopulation analysis of same-side crashes, right-seated passengers had an increased mortality (52.7% vs. 43.2%, *p* < 0.01) compared to left-seated passengers (OR = 1.55, 1.02, 2.36). Vehicle side crash safety ratings, available for 27.7% (*n* = 172) of same-side crashes, were not predictive of mortality.

**Conclusions:**

Except for same-side crashes, seat belts were associated with significantly lowered mortality. Despite this, seat belt wearing was low and represents one of several areas where further improvements in mortality might be realized.

## Background

Motor vehicle crashes are a leading cause of injury and death in the United States with nearly 46,000 deaths occurring in occupants of four-wheeled passenger vehicles during the 2-year time frame of this study (NHTSA 2012; Beck and West 2011). Historically, traveling in the front seat has been recognized as higher risk than in rear seats, and as such, a majority of the research has focused on improving the safety of front seat passengers (Berg et al. 2000; Evans and Frick 1988; Smith and Cummings 2006; Smith and Cummings 2004; Mayrose and Priya 2008). A result of the front seat focus is that much of the literature on rear-seated passengers has been on the relative safety of the rear seats compared to the front seats (Evans and Frick 1988; Smith and Cummings 2006; Smith and Cummings 2004; Mayrose and Priya 2008) or on restraint of children in rear seats (Berg et al. 2000; Howard et al. 2004; Lennon et al. 2008).

There are reports of the potential risk that unbelted rear-seated passengers pose to those in the front seat (Broughton 2004; Ichikawa et al. 2002; Mayrose et al. 2005; Shimamura et al. 2005), but factors that pose a mortality risk in adult rear-seated passengers themselves have not been fully explored (Mayrose and Priya 2008; Zhu et al. 2007). Research on rear-seated passengers has shown that belted rear-seated passengers have a lower risk of death than unbelted passengers (Evans and Frick 1988; Smith and Cummings 2006; Mayrose and Priya 2008; Zhu et al. 2007; Bodiwala et al. 1989). Some of the research on rear-seated passengers involved in side impacts includes seating position, but does not specifically distinguish whether the point of vehicle impact was on the same side or the opposite side of the rear-seated passenger, a potentially important predictor of fatality (Mayrose and Priya 2008; Zhu et al. 2007). Although not specific to rear-seated adult passengers, previous studies have noted that same-side (near-side) impacts have an increased risk of mortality for drivers and passengers compared to opposite-side (far-side) impacts; however, these studies did not consider passenger age and were performed on older vehicles (Fildes 2000; Laberge-Nadeau et al. 2009).

Recent studies have suggested that improvements to front seat safety may have outpaced that of rear-seated passengers (Bilston et al. 2010; Sahraei et al. 2010). Although there are reports that front-seat-occupant crash-related mortality has decreased with the use of newer vehicle models (Ryb et al. 2009, 2011; Brown and Bilston 2014), few studies have addressed the role of vehicle side crash safety ratings for rear-seated adult passengers (Teoh and Lund 2011). Further elucidation of these issues may identify areas of intervention to improve injury and mortality in this population.

The aims of this study are to examine rear-seated adult passenger mortality with respect to 1) select driver, passenger, vehicle, and crash characteristics hypothesized to be important to mortality; 2) the contribution of side crashes to mortality; and 3) whether and to what extent crash mortality is mitigated by seat belt status and side crash test ratings.

## Methods

### Data source

Data obtained from the Fatality Analysis Reporting System (FARS) for the calendar years 2010 to 2011 is made available by the National Highway Traffic Safety Administration (NHTSA) through download from a public FTP site. FARS contains vehicle-, person-, and crash-level variables for all fatal vehicle crashes occurring on a US roadway. Publicly available Insurance Institute for Highway Safety (IIHS) vehicle safety ratings for side crashes were programmed into the FARS dataset. Such vehicles receive a grade based upon a number of factors including passenger compartment infringement (IIHS 2014).

### Study population

Of the 17,701 rear-seated passengers involved in a fatal collision in 2010 to 2011, 7,998 were aged 18 or older and traveling in a four-wheeled passenger vehicle (van, sports utility vehicle (SUV), sedan, convertible, or station wagon) manufactured after 1970. Persons being transported in busses, large trucks, ATVs, farm equipment, motor homes, motorcycles, large limousines, emergency vehicles, straight trucks, and vehicles of unknown type were excluded from analysis. Rear-seated passengers missing belt status (*n* = 728) or with unknown mortality status (*n* = 41) were excluded, leaving a study population of 7,229.

### Variable classification

#### Person-level variables

##### Mortality

The primary outcome is mortality of a rear-seated adult occupant within 30 days of the crash from effects attributable to the crash.

##### Belt status

The primary exposure of interest is the belt status of adult rear-seated passengers. Belt status was analyzed as a dichotomous exposure with the use of any type of belt (i.e., lap belt only (*n* = 316), shoulder belt only (*n* = 19), or both (*n* = 3,134)) categorized as restrained.

##### Population age and gender of the driver and passenger

Age and gender were examined for both the driver and rear-seated passengers. Gender was categorized as male, female, or unknown. Driver age was categorized into an ordinal variable with the two youngest age ranges being drivers under 16 years of age and 16 to 19 years. Age of adult rear-seated passengers ranged from 18 to 100 years with the youngest category being ages 18 to 19. Both drivers and passengers aged 20 and older were categorized in 10-year intervals.

##### Alcohol and drug use

Driver alcohol and drug use was analyzed as a single dichotomous variable with the driver considered positive for alcohol or drugs if police or law enforcement reported alcohol or any drug involvement or if the driver was found to have a blood alcohol concentration of 0.01 or higher.

##### Previous driver violations

Previous moving violations by the driver were assessed using dichotomous variables for speeding, driving while intoxicated, or other moving violations occurring within the last 3 years.

##### Vehicle characteristics

Vehicle year was examined using two methods of categorization. The first was as a continuous variable ranging from 1970 to 2012. The second reflecting the introduction of vehicle safety improvements: 1970 to 1993, 1994 to 1997, 1998 to 2004, 2005 to 2008, and 2009 to 2012 (Ryb et al. 2011). In all categorizations, older vehicle model years (1970 to 1993 or 1970 to 1980) were used as the reference category. Four-wheeled passenger vehicles were categorized into six categories: convertible, station wagon, sedan, SUV, van, and pickup truck. Vehicle weight was categorized into six categories of increasing curb weight in pounds: less than 2,949; 2,950 to 3,549; 3,550 to 3,949; 3,950 to 4,449; 4,450 to 5,999; and greater than 6,000.

#### Crash-level characteristics

##### Point of impact/passenger seating

Point of impact in relation to passenger seating position was examined using a derived variable that integrated both seating position and the initial impact point in relation to the passenger. In addition, a determination was made as to whether the impact or most of the damage occurred on the same side as the rear-seated passenger using the initial point of impact/place of most damage to the vehicle and seating position information. The initial point of impact was delineated as being to the 1) front of the vehicle, 2) rear of the vehicle, 3) same side as the seated passenger, 4) opposite side of the seated passenger, 5) either side of the vehicle for middle-seated passengers, 6) non-collision (such as a rollover), or 7) underside of the vehicle. For multivariable modeling purposes, the point of initial impact was considered mutually exclusive. Front crashes were used as the reference category in the logistic and multilevel regressions.

##### Rollover, ejection, and speed

Vehicle rollover was a dichotomous variable, with any type of rollover (tripped, untripped, or unknown cause) categorized as a rollover. Ejection from the vehicle was analyzed as a three-level categorical variable: 1) not ejected, 2) partially ejected, or 3) fully ejected.

Speed of the vehicle was dichotomous with excessive speed characterized as present if investigators or law enforcement determined that the speed of the vehicle was excessive for road conditions, racing was involved, or if the driver was reported to be traveling above the posted speed limit. Travel speed itself would be preferable but was missing or not reported for over half of the sample.

##### Weekday/weekend

The potential association of social weekend (Friday night to Sunday afternoon) versus other times was examined as a dichotomous variable (Carpenter and Pressley 2013).

##### Weather

To assess the potential association of rear-seated passenger mortality and weather conditions, a single dichotomous variable was used to capture conditions related to rain, snow, fog, or wind.

##### Lighting

Light conditions were included as a three-level categorical variable classified as light, twilight, or dark.

#### Vehicle safety rating and subset analysis

A subset analysis was performed for vehicles with rear-seated passengers involved in same-side crashes (*n* = 621). Vehicles were categorized by their overall side crash test rating as ‘good’ , ‘acceptable’ , ‘marginal’ , ‘poor’ , or ‘unrated’ by matching FARS data on make, model, and year of vehicles to IIHS side crash test ratings (IIHS 2014). We report univariable relationships; however, for many analytical models, marginal and poor categories were collapsed due to small cell sizes. The side crash test ratings were determined by damage to the vehicle and crash test dummies in crash tests where the vehicle is struck by a barrier at a 90° angle traveling at 31 miles per hour (IIHS 2014). For the subpopulation analysis, vehicle year was restricted to vehicles manufactured after 1997, the earliest vehicle models for which side crash test ratings were available.

#### Statistical analysis

Bivariable associations between mortality and potential covariates were examined for categorical variables using the chi-square test or Fisher’s exact test for small expected cell sizes and Student *t*-tests for continuous variables. Univariable and multilevel multivariable logistic regressions were employed to investigate unadjusted and adjusted odds ratios (ORs) with 95% confidence intervals. The variables selected for investigation were those previously reported or hypothesized to be important factors in rear-seated adult passenger mortality. All final models were age and gender adjusted. Other variables which were found not to be significant predictors of mortality in univariable analysis or after adjustment for restraint use were not included in the final model. A final multilevel model was used to account for the potential hierarchical structure of the data, similar to previous studies of vehicle crashes that accounted for the hierarchical nature of crash data (Jones and Jørgensen 2003; Gkritza and Mannering 2008; Kim et al. 2007). A multilevel model was generated to control for violations of the assumption of independence (multiple rear-seated passengers traveling in the same vehicle) through the use of the GLIMMIX procedure, with the group-level variable VIN number, to adjust for clustering of passengers in vehicles.

In addition to the multilevel models for all rear-seated adult passengers, a subset analysis of passengers seated on the impact side of the vehicle examined the relationship between side crash test ratings and mortality. All analyses were performed in SAS 9.4.

## Results

The study population consisted of 7,229 adult rear-seated passengers aged 18 and older with a mean of 1.5 rear-seated passengers aged 18 or older per vehicle. Of those, 2,091 (28.9%) died within 30 days of a crash. Passenger characteristics, vehicle and crash characteristics, and unadjusted and adjusted multilevel models are shown in Tables 1, 2, and 3, respectively.

**Table 1:** **Person-level characteristics for rear-seated passengers, stratified by belt and passenger status (** ***n*** **, %)**

	**Belted**		**Unbelted**		**Total**	
	**Lived**	**Died**	**Lived**	**Died**		**Chi-square** ^**a**^
	***n*** **(%)**	***n*** **(%)**	***n*** **(%)**	***n*** **(%)**		***χ*** ^**2**^ **(** ***p*** **value)**
Total	2,918 (40.4)	560 (7.8)	2,220 (30.7)	1,531 (21.2)	7,229	536.2 (<0.0001)
Passenger characteristics						
Passenger age (years)						233.8 (<0.0001)
<20	492 (16.9)	69 (12.3)	474 (21.4)	257 (16.8)	1,292 (17.9)	
20 to 44	1,652 (56.6)	182 (32.5)	1,149 (65.3)	884 (57.7)	4,167 (57.6)	
45 to 64	510 (17.5)	117 (20.9)	239 (10.8)	240 (15.7)	1,106 (15.3)	
≥65	264 (9.1)	192 (34.3)	58 (2.6)	150 (9.8)	664 (9.2)	
Passenger gender						2.3 (0.022)
Male	1,518 (52.0)	228 (40.7)	1,327 (59.8)	868 (56.7)	3,941 (54.5)	
Driver characteristics						
Driver age (years)						15.9 (0.0435)
<20	353 (12.1)	64 (11.4)	342 (15.5)	243 (15.9)	1,002 (13.9)	
20 to 44	1,571 (54.0)	240 (42.9)	1,421 (64.2)	943 (61.8)	4,175 (57.9)	
45 to 64	713 (24.5)	163 (29.1)	345 (15.6)	256 (16.8)	1,477 (20.5)	
≥65	275 (9.4)	93 (16.6)	106 (4.8)	84 (5.5)	558 (7.7)	
Driver gender						1.9 (0.1643)
Male	2,037 (70.0)	368 (65.7)	1,633 (74.0)	1,092 (71.7)	5,130 (71.2)	
Driver drinking or drugged						21.3 (<0.0001)
Yes	492 (17.6)	94 (18.1)	850 (40.6)	553 (38.4)	1,989 (29.0)	
Driver belt status						2.1 (0.1469)
Belted	2,585 (90.5)	513 (94.8)	1,131 (52.8)	941 (64.5)	5,170 (73.9)	

^a^Chi-square and *p* value expressed are for the relationship between the left-hand variable in question and death.

**Table 2:** **Crash- and vehicle-level characteristics, stratified by belt use and rear-seated passenger mortality**

	**Belted**		**Unbelted**		**Total**	
	**Lived**	**Died**	**Lived**	**Died**		**Chi-square** ^**a**^
	***n*** **(%)**	***n*** **(%)**	***n*** **(%)**	***n*** **(%)**		***χ*** ^**2**^ **(** ***p*** **value)**
Total	2,918 (40.4)	560 (7.8)	2,200 (30.7)	1,531 (21.2)	7,229	
Vehicle characteristics						
Vehicle make year						10.4 (0.0340)
<1994	148 (5.1)	29 (5.2)	173 (7.8)	128 (8.4)	478 (6.6)	
1994 to 1997	347 (11.9)	76 (13.6)	376 (16.9)	261 (17.1)	1,060 (14.7)	
1998 to 2004	1,311 (44.9)	216 (38.6)	1,170 (52.7)	745 (48.7)	3,442 (47.6)	
2004 to 2008	776 (26.6)	163 (29.1)	401 (18.1)	310 (20.3)	1,650 (22.8)	
2009 to 2012	336 (11.5)	76 (13.6)	100 (4.5)	87 (5.7)	599 (8.3)	
Curb weight (lbs)						120.9 (<0.0001)
<2,949	417 (14.6)	119 (21.9)	359 (16.5)	283 (19.0)	1,178 (16.7)	
2,950 to 3,549	629 (22.0)	187 (34.4)	503 (23.1)	430 (28.8)	1,749 (24.8)	
3,550 to 3,949	267 (9.3)	57 (10.5)	234 (10.8)	157 (10.5)	715 (10.1)	
3,950 to 4,449	352 (12.3)	55 (10.1)	232 (10.7)	160 (10.7)	799 (11.3)	
4,450 to 5,999	618 (21.6)	79 (14.6)	393 (18.1)	265 (17.8)	1,355 (19.2)	
6,000 or more	576 (20.2)	46 (8.5)	454 (20.1)	196 (13.2)	1,272 (18.0)	
Model type						119.0 (<0.0001)
Convertible	10 (0.3)	0 (0.0)	5 (0.2)	8 (0.5)	23 (0.3)	
Station wagon	76 (2.6)	22 (3.9)	24 (1.1)	26 (1.7)	148 (2.1)	
Sedan	1,097 (37.6)	329 (58.8)	918 (41.4)	758 (49.5)	3,102 (42.9)	
SUV	834 (28.6)	102 (18.2)	656 (29.6)	436 (28.5)	2,028 (28.1)	
Van	466 (16.0)	71 (12.7)	299 (13.5)	141 (9.2)	977 (13.5)	
Pickup truck	435 (14.9)	36 (6.4)	318 (14.3)	162 (10.6)	951 (13.5)	
Crash characteristics						
Rollover						42.9 (<0.0001)
Yes	682 (23.4)	134 (23.9)	1,049 (47.3)	741 (48.4)	2,606 (36.1)	
Ejected						843.3 (<0.0001)
Fully	19 (0.7)	18 (0.7)	489 (22.2)	706 (46.5)	1,232 (17.1)	
Partially	4 (0.1)	14 (2.5)	44 (2.0)	105 (6.9)	167 (2.3)	
Not ejected	2,891 (99.2)	527 (94.3)	1,666 (75.8)	707 (46.6)	5,791 (80.5)	
Excessive speed						31.8 (<0.0001)
Yes	550 (19.0)	119 (21.7)	799 (36.6)	562 (37.5)	2,030 (28.5)	
Side						8.1 (0.0171)
Right	1,440 (49.9)	299 (53.4)	921 (42.4)	737 (49.3)	3,397 (47.8)	
Left	1,202 (41.7)	224 (40.0)	820 (37.8)	536 (35.9)	2,782 (39.1)	
Middle	243 (8.4)	37 (6.6)	429 (19.8)	222 (14.8)	931 (13.1)	
Initial point of impact						171.6 (<0.0001)
Front	1,661 (58.3)	257 (47.1)	1,167 (54.2)	646 (43.6)	3,731 (53.1)	
Back	354 (12.4)	87 (15.9)	110 (5.1)	132 (8.9)	683 (9.7)	
Opposite side	221 (7.8)	43 (7.9)	160 (7.4)	86 (5.8)	510 (7.3)	
Same side	183 (6.4)	93 (17.0)	121 (5.6)	197 (13.3)	594 (8.5)	
Middle-seat side collision	34 (1.2)	8 (1.5)	61 (2.8)	42 (2.8)	145 (2.1)	
No collision	304 (10.7)	45 (8.2)	447 (20.8)	320 (21.6)	1,116 (15.9)	
Undercarriage	90 (3.2)	13 (2.4)	86 (4.0)	58 (3.9)	247 (3.5)	

^a^Chi-square and *p* value expressed are for the relationship between the left-hand variable in question and death. Vehicle model year (continuous) is the OR for a 1-year increase.

**Table 3:** **Odds ratios (with 95% CIs) for mortality using unadjusted, adjusted, and multilevel multivariable logistic regression**

	**Unadjusted mortality**	**Belt status adjusted mortality**	**Multivariable multilevel mortality**
Passenger belted	0.28 (0.25, 0.31)	-	0.33 (0.28, 0.39)
Passenger age (years)			
18 to 19	Ref	Ref	Ref
20 to 29	0.99 (0.86, 1.16)	0.97 (0.83, 1.14 )	1.17 (0.95, 1.46)
30 to 39	1.05 (0.87, 1.27)	1.14 (0.94, 1.40 )	1.71 (1.32, 2.21)
40 to 49	1.27 (1.04, 1.57)	1.40 (1.13, 1.74 )	2.28 (1.72, 3.10)
50 to 59	1.27 (1.02, 1.59)	1.67 (1.32, 2.11 )	3.00 (2.21, 4.06)
60 to 69	1.87 (1.47, 2.38)	3.06 (2.36, 3.97 )	6.53 (4.67, 9.14)
70 to 79	2.61 (2.00, 3.41)	4.56 (3.41, 6.10 )	8.98 (6.15, 13.11)
Over 80	5.67 (4.16, 7.72)	11.09 (7.96, 15.45 )	26.68 (17.55, 40.56)
Passenger male	0.89 (0.80, 0.98)	0.79 (0.71, 0.88)	0.96 (0.84, 1.09)
Rear seat position			
Middle	Ref	Ref	
Right	1.14 (0.97, 1.34)	1.50 (1.27. 1.77)	
Left	0.98 (0.83, 1.15)	1.27 (1.07, 1.51)	
Driver age			
<16	1.41 (0.91, 2.18)	1.45 (0.92, 2.29)	1.35 (0.77, 2.35)
17 to 19	Ref	Ref	Ref
20 to 29	0.99 (0.84, 1.16)	0.96 (0.81, 1.14)	0.81 (0.65, 1.01)
30 to 39	0.83 (0.68, 1.01)	0.90 (0.73, 1.10)	0.71 (0.54, 0.93)
40 to 49	0.87 (0.71, 1.07)	0.99 (0.80, 1.23)	0.61 (0.45, 0.81)
50 to 59	0.95 (0.77, 1.18)	1.23 (0.98, 1.54)	0.71 (0.52, 0.97)
60 to 69	0.97 (0.76, 1.24)	1.34 (1.04, 1.73)	0.59 (0.41, 0.83)
70 to 79	0.94 (0.70, 1.28)	1.42 (1.03, 1.95)	0.37 (0.24, 0.58)
Over 80	1.45 (1.01, 2.41)	2.35 (1.49, 3.72)	0.34 (0.24, 0.62)
Driver drinking or drugged	1.30 (1.16, 1.46)	0.94 (0.83, 1.06)	
Vehicle model year (continuous)^a^	0.99 (0.98, 1.00)	1.01 (1.00, 1.02)	
Vehicle model year^b^			
<1994	Ref	Ref	
1994 to 1997	0.94 (0.74, 1.18)	0.99 (0.78, 1.25)	
1998 to 2004	0.78 (0.64, 0.96)	0.85 (0.69, 1.06)	
2005 to 2008	0.81 (0.65, 1.01)	1.05 (0.84, 1.32)	
2009 to 2012	0.75 (0.58, 0.98)	1.16 (0.88, 1.52)	
Model type			
Sedan	Ref	Ref	Ref
Pickup	0.49 (0.41, 0.58)	0.48 (0.40, 0.58)	0.86 (0.59, 1.25)
SUV	0.67 (0.59, 0.76)	0.65 (0.57, 0.74)	0.64 (0.50, 0.81)
Van	0.51 (0.43, 0.61)	0.55 (0.46, 0.65)	0.62 (0.46, 0.84)
Convertible	1.00 (0.42, 2.34)	0.96 (0.39, 2.35)	0.60 (0.19, 1.90)
Station wagon	0.89 (0.63, 1.27)	1.16 (0.81, 1.68)	1.01 (0.64, 1.59)
Vehicle curb weight (lbs)			
<2,949	Ref	Ref	Ref
2,950 to 3,549	1.05 (0.90, 1.23)	1.07 (0.91, 1.26)	1.11 (0.91, 1.35)
3,550 to 3,949	0.83 (0.68, 1.01)	0.81 (0.66, 1.00)	0.89 (0.67, 1.17)
3,950 to 4,449	0.71 (0.58, 0.87)	0.74 (0.60, 0.91)	0.84 (0.62, 1.14)
4,450 to 5,999	0.66 (0.55, 0.78)	0.69 (0.57, 0.82)	0.80 (0.58, 1.10)
6,000 or more	0.45 (0.38, 0.55)	0.45 (0.37, 0.54)	0.52 (0.36, 0.77)
Not ejected	Ref	Ref	Ref
Ejected fully	5.26 (4.62, 5.99)	3.49 (3.03, 4.02)	4.86 (4.07, 5.81)
Ejected partially	9.15 (6.50, 12.86)	6.54 (4.63, 9.25)	8.46 (5.71, 12.52)
Initial impact point			
Front	Ref	Ref	Ref
Back	1.48 (1.24, 1.77)	1.85 (1.53, 2.23)	1.83 (1.46, 2.30)
Middle seat, side crash	1.65 (1.16, 2.34)	1.29 (0.89, 1.85)	1.28 (0.84, 1.97)
Opposite side	1.06 (0.86, 1.31)	1.07 (0.86, 1.34)	0.84 (0.65, 1.10)
Same side	2.99 (2.50, 3.57)	3.11 (2.58, 3.75)	2.76 (2.22, 3.44)
Undercarriage	1.26 (0.95, 1.68)	1.13 (0.84, 1.52)	1.06 (0.74, 1.50)
Non-collision	1.53 (1.32, 1.77)	1.22 (1.05, 1.42)	1.03 (0.84, 1.25)
Rollover	1.42 (1.28, 1.57)	1.04 (0.93, 1.16 )	
Excessive speed	1.38 (1.23, 1.54)	1.07 (0.96, 1.21)	1.25 (1.07, 1.45)

^a^Vehicle model year (continuous) is the OR for a 1-year increase. ^b^Vehicle model year was considered as both continuous and categorical; in adjusted models, it was adjusted for once.

### Passenger characteristics

#### Passenger age

The majority of adult rear-seated passengers involved in fatal crashes were between the ages of 18 and 29 (*n* = 4,140, 57.3%). Elderly passengers aged 65 and older (*n* = 664) comprised fewer than 10% of the study population, but 16.4% of total mortality. Approximately half of the passengers aged 65 and older died (*n* = 342, 51.5%) (Table 1), but this ranged from 41.5% in 65- to 69-year-olds to 65.7% of those aged 80 years and older.

#### Passenger gender

A higher proportion of male than female passengers were involved in fatal crashes, but a higher proportion of female passengers died (30.3% vs. 27.8%, *p* = 0.02).

### Driver characteristics

#### Driver age

Half of the drivers involved in fatal crashes with a rear-seated adult passenger were under the age of 30 (*n* = 3,624, 50.4%). Younger drivers were more likely to have younger rear-seated passengers, with 44.2% (*n* = 572) of teen passengers traveling with a teen driver. In the fully adjusted model, driver age was significantly associated with rear-seated passenger mortality with drivers under 30 years of age having increased odds of rear-seated passenger mortality (Table 3).

#### Driver gender

The majority of drivers involved in fatal crashes were male (71.2%) (Table 1). However, driver gender was not associated significantly with rear-seated passenger mortality.

#### Alcohol and drug presence

More than a quarter of drivers (29.0%) involved in fatal crashes were drinking or drugged at the time of the crash (Table 1). The presence of alcohol and/or drugs in drivers was associated with an unadjusted increase in rear-seated mortality (OR 1.30, 95% CI 1.16, 1.46), but not in adjusted multilevel models (Table 3).

#### Previous moving violations

Previous driver convictions for speeding, driving while intoxicated (DWI), or total moving violations within the last 3 years were not associated with rear-seated passenger mortality (*p* > 0.05).

### Belt status and mortality

**Figure 1:**
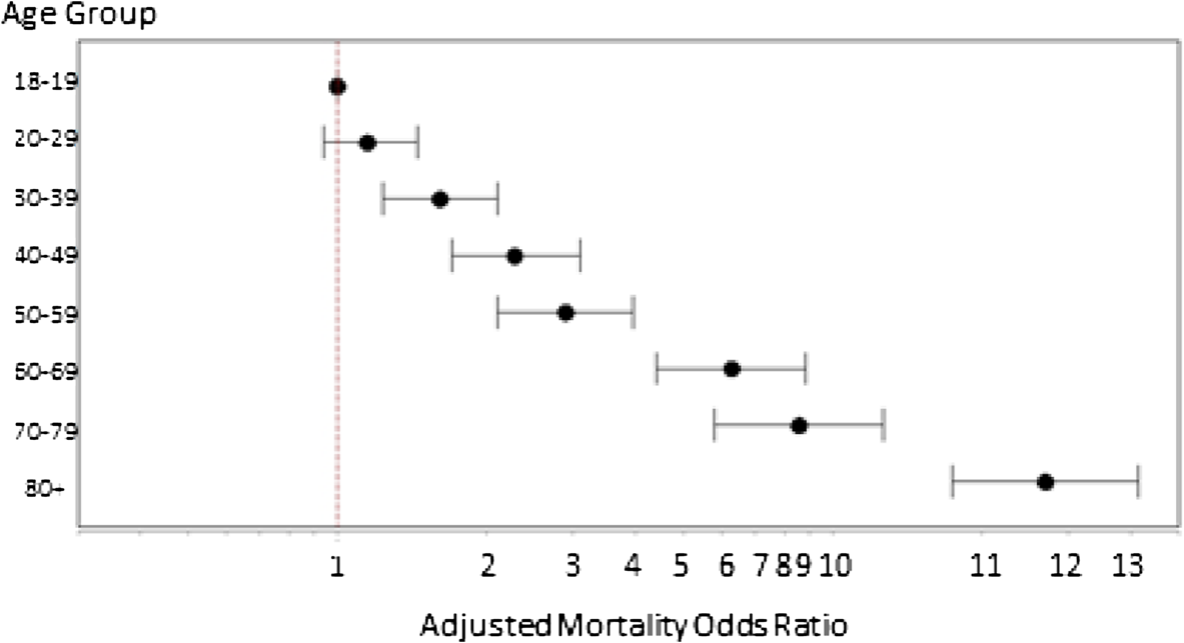
**Rear passenger adjusted mortality by age group (odds ratios with 95% confidence intervals).** Odds ratios are adjusted for passenger gender, belt status, and ejection; driver age and alcohol/drug status; vehicle model year, type, and curb weight; and crash characteristics for point of impact, rollover, and excessive speed. The reference group is 18- to 19-year-olds.

Unbelted passengers were approximately 3.5 times more likely to die in crashes than belted passengers. In univariable analysis, being unbelted in the rear seat of the vehicle more than tripled the odds of death (OR = 3.60, 95% CI 3.22, 4.02), but this varied by age of the passenger (Table 3). The impact of belt status on mortality in elderly occupants was higher than that in younger aged passengers, particularly in multilevel multivariable models (Table 3). Although mortality increased with each decade of passenger age, this increase accelerated after age 60. Age increases were observed consistently across unadjusted, restraint-adjusted, and multilevel models with the largest age impacts noted for those aged 80 and over (Figure 1).

**Figure 2:**
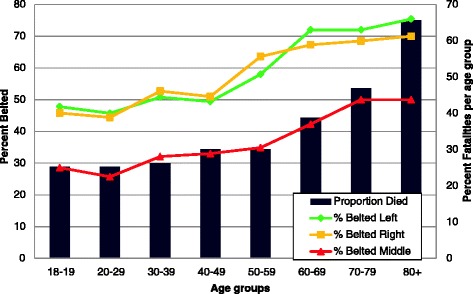
**Mortality and belt status by seating position and age group.** The left *y*-axis indicates the proportion belted, the right *y*-axis indicates the proportion who died, and the horizontal *x*-axis indicates the age groups. Percent mortality (black bars) and belt status by seating position (lines) is shown by age group.

Driver belt status was strongly predictive of passenger belt status with passengers more than seven times more likely to be belted when the driver was belted (OR = 7.62, 95% CI 6.65, 8.73). However, driver belt status was not predictive of rear-seated passenger mortality in univariable analyses (*p* = 0.15). Older passengers were more likely to be belted, traveling with a driver who was belted, and were also more likely to die in the crash (Figure 2). Passengers aged 80 years and older rode with drivers who were almost always belted. In contrast, drivers with 18- to 19-year-old passengers were less frequently belted (67.9% vs. 94.3%, *p* < 0.0001).

### Vehicle characteristics

#### Model year

Vehicle year, considered both continuously and categorized by year of introduction of major vehicle safety improvements, was significantly associated with lower rear-seated passenger mortality in unadjusted models, with 25% lower mortality in the newest vehicles (2009 to 2012) compared to those manufactured prior to 1994 (Table 3). Vehicle year, measured in decades, was not a significant predictor of mortality. Once adjusted for belt use, vehicle year was no longer significantly predictive of mortality (*p* = 0.17).

#### Weight and model

Vehicle weight and model type were each significant predictors of mortality with larger and heavier vehicles, such as SUVs or vans, showing a protective effect (Table 3). In unadjusted analyses, passengers seated in SUVs had 33% lower mortality, and those seated in vans had a 49% lower mortality than sedans. In adjusted analyses, SUVs were associated with a 36% reduction and vans were associated with a 38% reduction in mortality, compared to sedans. Vehicles weighing 6,000 lbs or more were associated with lower mortality (Table 3).

### Crash characteristics

#### Passenger rear seating positions

Nearly half (47.8%) of rear-seated passengers were seated on the right side (opposite driver), with 39.1% seated on the left behind the driver and 13.1% in the middle-seat position (Table 2).

#### Seating position and belt status

Passenger belt status differed by seating position with more than two thirds (69.5%) of middle-seated passengers being unbelted compared to the left and right seating positions where about half (48.8%) were unrestrained. Younger passengers were more likely to be in the middle-seat position than older rear-seated passengers.

#### Mortality differentials by seating position

Outer seated passengers accounted for nearly 90% of all rear-seated passenger deaths, with about half of all deaths being seated on the right side and more than one third on the left (Table 2).

#### Point of impact by passenger seat position

The initial impact point with the highest mortality for rear-seated passengers was an impact to the same side of the vehicle as the passenger was seated. The initial impact point with the lowest mortality for rear-seated passengers was a frontal crash. Passengers involved in crashes to the rear of the vehicle showed a statistically significant increase in odds of mortality compared to frontal crashes. Opposite-side impacts carried no additional risk compared to frontal crashes (Table 3).

**Figure 3:**
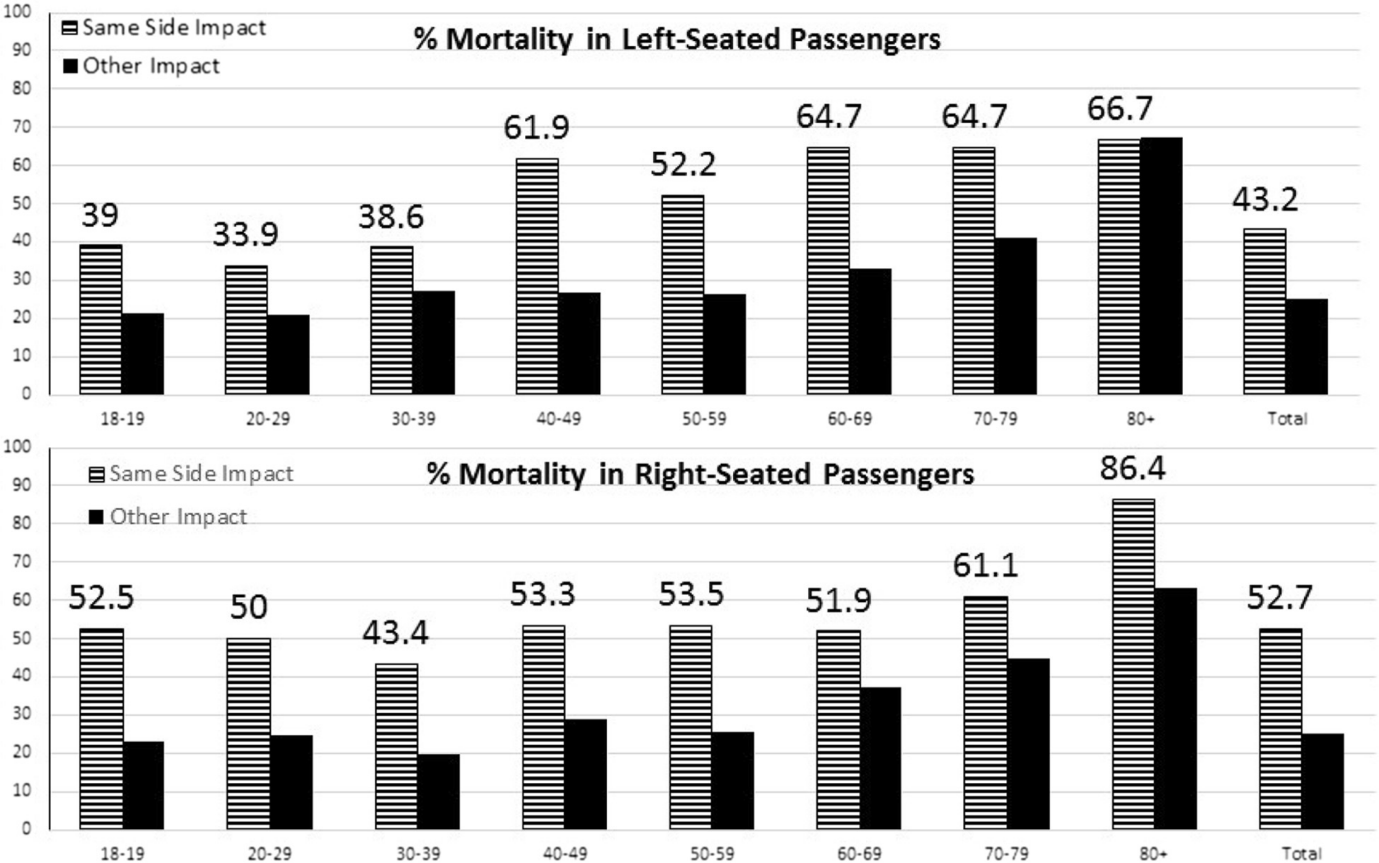
**Same-side passenger deaths stratified by age and same-side crash (left vs. other) and (right vs. other).** Percent mortality is shown for same-side crashes (striped) and other crashes (black) by passenger age.

In multivariable models, same-side impacts were associated with a nearly threefold increase in mortality (OR = 2.76, 95% CI 2.22, 3.44) (Table 3) compared to frontal crashes. Among passengers seated on the impact side, mortality was higher for right- versus left-seated passengers (52.7% vs. 43.2%, *p* < 0.01), though this effect varies by age group (Figure 3). In a multivariable subpopulation analysis of same-side crashes adjusted for all covariates, sitting on the right side was associated with an increase in mortality compared to the left side (OR = 1.55, 95% CI 1.02, 2.36). Adjustment for belt status did not appear to affect the odds of mortality from same-side impacts.

For middle-seated passengers, unadjusted analyses during a side collision showed a significantly higher odds of mortality compared to frontal crashes (OR = 1.65, 95% CI 1.16, 2.34). However, only 30.2% of middle-seated passengers were restrained. Adjusting for passenger belt status reduced the odds of death in side collisions for middle-seated passengers (OR = 1.29, 95% CI 0.89, 1.85) (Table 3).

#### Excess speed

Excess vehicle speed was associated with increased mortality in rear-seated passengers (OR = 1.38, 95% CI 1.23, 1.54) in both unadjusted and adjusted analyses (Table 3).

#### Rollovers and ejections

Rollovers occurred in 36.1% and ejections in 19.5% of crashes (Table 2). Restrained rear-seated passengers involved in a rollover were 96% less likely to be ejected and 72% less likely to die than unbelted passengers. In unadjusted models, compared to no ejection, partial ejections had the highest mortality (OR = 9.15, 95% CI 6.50, 12.86) followed by total ejections (OR = 5.26, 95% CI 4.62, 5.99) (Table 2).

#### Environmental factors

Weather, light conditions, day of the week, and time of day were not associated with rear-seated adult mortality.

### Independent predictors of mortality

In multilevel, multivariable models that adjusted for the clustering of rear occupants traveling in the same vehicles, rear passenger restraint use was associated with a 67% reduction in total mortality (Table 3). Protective effects were noted for larger and heavier vehicles, with vehicles over 6,000 lbs reducing mortality by 48.0% (Table 3). Predictors of increased mortality were advancing passenger age, younger driver age, excessive speed, ejection, rear impact, and same-side crash (Table 3). In the adjusted model, driver alcohol and drug use, rollover, passenger gender, and vehicle year were not predictive of mortality.

Compared to passengers aged 18 to 19 years, older passengers experienced increasing mortality with each decade of age. Passengers aged 80 and older were 26.7 times more likely to die as a result of the crash than 18- to 19-year-olds (Table 3).

### Subpopulation analysis with safety ratings

#### Vehicle safety ratings and rear-seated passenger mortality

The subgroup analysis of same-side crashes included 621 rear-seated passengers traveling in vehicles manufactured after 1997, of which, 431 (69.4%) passengers were traveling in a vehicle that did not rollover. Only 27.7% (*n* = 172) of vehicles involved in same-side crashes had an IIHS side safety rating. Vehicle ratings by rear-seated passenger mortality are shown in Table 4. Rear-seated passenger mortality by rating was 57.9% for good-rated vehicles, 47.8% for acceptable-rated vehicles, 46.2% for those marginal-rated vehicles, 55.0% for poor-rated vehicles, and 45.0% for unrated vehicles. For those where it was available, vehicle side crash test ratings (with collapsed marginal and poor categories) were not associated with rear-seated adult passenger mortality in either unadjusted or adjusted models.

**Table 4:** **Side crash test ratings for passengers involved in same-side crashes, stratified by rear-seated mortality for vehicle models 1997 to 2012**

	**Lived,** ***n*** **(%)**	**Died,** ***n*** **(%)**	**Total,** ***n*** **(%)**	**Chi-square** ^**a**^
	**325 (52.3)**	**296 (47.7)**	**621 (100)**	***χ*** ^**2**^ **(** ***p*** **value)**
Rating				5.8 (0.2159)
Good	32 (9.9)	44 (14.9)	76 (12.2)	
Acceptable	12 (3.7)	11 (3.7)	23 (3.7)	
Marginal	7 (2.2)	6 (2.0)	13 (2.1)	
Poor	27 (8.3)	33 (11.2)	60 (9.7)	
Total rated	78 (24.0)	94 (31.8)	172 (27.7)	
Unrated	247 (76.0)	202 (68.2)	449 (72.3)	

^a^Chi-square and *p* value expressed are for the relationship between rating (including unrated) and mortality. This includes all side crash test ratings (rollover and non-rollovers). Sensitivity analyses were performed with and without rollovers, but did not change the findings.

## Discussion

Among those involved in a fatal collision, rear-seated passengers who wore a seat belt were approximately one third as likely to die, although this effect differed across passenger age, seating positions, and point of crash impact. Despite reports that the rear middle seat confers a protective effect compared to other seating positions, this was offset in our study by differences in belt wearing by seating position with more than two thirds of middle rear-seated passengers being unbelted. Of all the modifiable predictors of mortality, belt use was a highly important protective factor, a finding consistent with previous studies (Beck and West 2011; Mayrose and Priya 2008; Zhu et al. 2007; Bodiwala et al. 1989). The use of seat belts significantly decreased the odds of death associated with ejections, rollovers, and most points of impact except same-side crashes. Same-side crashes were infrequent, comprising fewer than 10% of all crashes, but were highly fatal with neither belt status nor the current car safety rating system for rear-seated passengers conferring a significant benefit. Just over one quarter of the vehicles involved in same-side crashes had an IIHS safety rating.

Passenger seating position played an important role in passenger mortality. Passenger age was associated with seating position, as middle-seated passengers tended to be younger than outboard-seated passengers and were less likely to be wearing a seat belt. In adjusted models of all rear-seated passengers, mortality was lower for both middle- and left-seated compared to right-seated passengers. In addition, when examining same-side crashes, passengers seated on the right side had an increased odds of death compared to passengers seated on the left, after adjustment for a number of possible confounding factors. Further study is needed to determine whether this finding is explained by left turns across traffic exposing right-seated passengers to same-side crashes from faster moving oncoming traffic compared to left-seated passengers who may be less frequently exposed to high-speed turn-related crashes. Left-seated passengers exposed to right turns are less likely to be in the path of oncoming traffic.

The driver’s belt status did not independently predict rear-seated adult passenger mortality, but was highly predictive of the passenger’s belt status. This paradoxical finding was explained by belted drivers being more likely to transport older, high-risk passengers. Older passengers were more likely to be belted than younger passengers and were also more likely to die despite being belted. Younger drivers most often transported younger passengers. The finding of differential belt wearing in younger passengers suggests an area for much needed improvement.

Our finding that being belted did not reduce the odds of mortality in same-side crashes is not different from that of other findings in relation to side crashes, although these studies did not specifically address rear-seated passengers (Fildes 2000; Laberge-Nadeau et al. 2009). Further study is needed to assess whether mortality in belted passengers held in place during same-side impacts might be improved by strengthening vehicle engineering features or through rear passenger airbags. This finding suggests that, despite many vehicle safety improvements, the fleet of vehicles in which rear-seated adult passengers ride is an area for potential future improvement.

Larger and heavier vehicles showed a significant protective effect for rear-seated adult passengers. This is consistent with past studies of drivers and front-seated passengers, where it was also demonstrated that larger and heavier vehicles were associated with reduced mortality (NHTSA 1997; Evans and Frick 1993; Farmer et al. 1997). Excess vehicle speed at the time of the crash significantly increased the odds of dying for rear-seated passengers, possibly due to increased severity of crashes. Our attempt to examine vehicle side crash ratings was hampered by the small proportion of vehicles in our sample that were rated and yielded results that were inconsistent with our hypothesis. For the vehicles for which there were side crash test ratings available, these ratings were not predictive of mortality in adult rear-seated passengers involved in same-side crashes, even after taking into account possible confounding factors. Further study, with a larger sample size, is needed in order to better parse out these relationships.

This study had limitations. It analyzed a data set of rear-seated occupants involved in a fatal collision and may not be generalizable to all crashes. The dichotomous categorization of speeding was used due to data limitations related to large quantities of missing data on actual vehicle speed. This may not have fully captured the importance of speed. It is possible that the presence of alcohol and drugs is underestimated. It is possible to have misclassified belt status if it was inaccurately reported or recorded (Zhu et al. 2007). Striking vehicle characteristics, such as vehicle type, travel speed, and weight were not analyzed. These factors could be additional important predictors for rear-seated passenger mortality, as previous studies have shown that the difference in size between vehicles can impact mortality (NHTSA 1997; Evans and Frick 1993; Farmer et al. 1997). The effect of the total number of passengers per vehicle was not modeled. History of driving violations data was limited to drivers with convictions in the last 3 years, likely underestimating the number of drivers who had a history of driving violations. Analysis of same-side crashes was limited by both the relatively small number of vehicles involved in such crashes as well as the small proportion that had an IIHS rating. Furthermore, we did not have data on side air bag deployment which could have influenced mortality. Finally, although mortality was similar across most variables with missing data, this was not true of driver impairment where drivers with missing information tended to be driving older passengers.

## Conclusions

In conclusion, these findings suggest that additional work is needed to improve safety for rear-seated adult passengers, particularly older ones. It extends the body of knowledge on mortality in rear-seated adults by better elucidating the relationships among driver and passenger characteristics, belt status, seating position, point of impact, and crash mortality. Mortality associated with same-side impacts needs further investigation, particularly in regard to whether vehicle safety standards impact same-side mortality, the most fatal of crash impact points, which remained high even in belted passengers. The observation that nearly half of outer-seated passengers and more than two thirds of middle-seated occupants were not belted is a notable area for focused intervention. Except for same-side crashes, rear seat belt use was significantly associated with reduced mortality, a finding that may support passage and enforcement of rear seat belt laws, as currently only 17 states and the District of Columbia have primary rear seat belt laws covering the full age span.
